# CNNAttLSTM: an attention-enhanced CNN–LSTM architecture for high-precision jackfruit leaf disease classification

**DOI:** 10.3389/fpls.2025.1720471

**Published:** 2026-01-05

**Authors:** Gaurav Tuteja, Fuad Ali Mohammed Al-Yarimi, Amna Ikram, Rupesh Gupta, Ateeq Ur Rehman, Jeewan Singh, Irene Delgado Noya, Luis Alonso Dzul Lopez

**Affiliations:** 1 Chitkara University Institute of Engineering and Technology, Chitkara University, Punjab, India; 2 Applied College of Mahail Aseer, King Khalid University, Muhayil Aseer, Saudi Arabia; 3 Department of Computer Science and IT, Government Sadiq College Women University, Bahawalpur, Pakistan; 4 School of Computing, Gachon University, Seongnam-si, Republic of Korea; 5 Department of Mechanical Engineering Graphic Era (Deemed to be University), Clement town, Dehradun, India; 6 Universidad Europea del Atlántico, Santander, Spain; 7 Universidad Internacional Iberoamericana, Campeche, Mexico; 8 Universidade Internacional do Cuanza, Cuito, Bié, Angola; 9 Fundación Universitaria Internacional de Colombia, , Bogotá, Colombia; 10 Universidad Internacional Iberoamericana, Arecibo, Puerto Rico; 11 Universidad de La Romana, La Romana, Dominican Republic

**Keywords:** plant disease detection, jackfruit leaf classification, attention mechanisms, CNNAttLSTM model, precision agriculture, agricultural AI, disease diagnosis

## Abstract

**Introduction:**

Jackfruit cultivation is highly affected by leaf diseases that reduce yield, fruit quality, and farmer income. Early diagnosis remains challenging due to the limitations of manual inspection and the lack of automated and scalable disease detection systems. Existing deep-learning approaches often suffer from limited generalization and high computational cost, restricting real-time field deployment.

**Methods:**

This study proposes CNNAttLSTM, a hybrid deep-learning architecture integrating Convolutional Neural Networks (CNN), Long Short-Term Memory (LSTM) units, and an attention mechanism for multi-class classification of algal leaf spot, black spot, and healthy jackfruit leaves. Each image is divided into ordered 56×56 spatial patches, treated as pseudo-temporal sequences to enable the LSTM to capture contextual dependencies across different leaf regions. Spatial features are extracted via Conv2D, MaxPooling, and GlobalAveragePooling layers; temporal modeling is performed by LSTM units; and an attention mechanism assigns adaptive weights to emphasize disease-relevant regions. Experiments were conducted on a publicly available Kaggle dataset comprising 38,019 images, using predefined training, validation, and testing splits.

**Results:**

The proposed CNNAttLSTM model achieved 99% classification accuracy, outperforming the baseline CNN (86%) and CNN–LSTM (98%) models. It required only 3.7 million parameters, trained in 45 minutes on an NVIDIA Tesla T4 GPU, and achieved an inference time of 22 milliseconds per image, demonstrating high computational efficiency. The patch-based pseudo-temporal approach improved spatial–temporal feature representation, enabling the model to distinguish subtle differences between visually similar disease classes.

**Discussion:**

Results show that combining spatial feature extraction with temporal modeling and attention significantly enhances robustness and classification performance in plant disease detection. The lightweight design enables real-time and edge-device deployment, addressing a major limitation of existing deep-learning techniques. The findings highlight the potential of CNNAttLSTM for scalable, efficient, and accurate agricultural disease monitoring and broader precision agriculture applications.

## 1 Introduction

Jackfruit is highly susceptible to a plethora of diseases due to widespread planting, which harms the volume and quality of the fruit and the economic well-being of farming communities. These challenges in early disease detection, as seen, are attributed to the lack of automated disease detection technology and the forced use of manual detection methods that are mostly labour-intensive and subject to errors. Although some existing research has examined computer vision and classification algorithms for detecting fruit diseases, they have limited generalisation capabilities and are not capable of diagnosing all pathologies of jackfruits. An AI-based agro-medical system that combines computer vision and machine learning has high potential for diagnosing plant diseases, but additional tuning is needed to be applied in the field of precision agriculture (Habib et al., 2022). Deep learning has revolutionised the concept of plant health monitoring by addressing the limitations and inefficiencies of traditional manual inspections. Convolutional Neural Networks (CNNs) are among the methods that achieve better results in detecting plant diseases, especially when trained using transfer learning to attain peak performance accuracy.

Nevertheless, current models are often marred by a computational drawback that restricts their practicality for real-time applications. MobileNetV2, a high-performance and lightweight model, is an ideal alternative, as it enhances accuracy and scalability to automatically identify diseases in agricultural settings (Banarase and Shirbahadurkar, 2024). This is essential in reducing losses in yields and enhancing sustainable agriculture practices by detecting and managing plant disease conditions at an early stage. Eye-level inspection is labour-intensive and inaccurate. Deep Convolutional Neural Networks (DCNNs) are an effective method for obtaining high-precision image-based disease diagnosis. The current systems, nevertheless, are computationally heavy, thus restricting their uptake. Early disease detection can be achieved using new DCNN designs, which minimise agricultural losses and make yield production sustainable (Rajalakshmi et al., 2024). Accurate identification of banana leaf diseases will be a crucial element in preventing crop losses and promoting agricultural sustainability. Deep learning models, particularly CNNs, have enabled the automatic classification of diseases. Nevertheless, extracting features is challenging due to the presence of noisy images and similar symptoms. The hybrid and multi-scale feature learning techniques, along with the hybrid activation function, can enhance detection robustness and accuracy, thereby advancing disease detection in the field of agriculture (Deng et al., 2024). Skip connection CNNs enable further optimisation of disease-specific feature extraction, thereby increasing the detection rate. The majority of current models address the problem of macronutrient deficiency; however, recent advances in the field of deep learning enable the identification of micronutrient imbalances, allowing for the application of precision agriculture principles and effective nutrient management practices (Sunitha et al., 2024).

CNNs have been successfully applied to solve these diseases, as well as transfer learning, in mango leaves, thereby addressing some of the key challenges in precision agriculture. Nevertheless, the computational efficiency and extensive generalisation to different environmental conditions need further research (Pratap and Krishna, 2024). By combining DenseNet-121 and VGG19 with PSO, the classification performance can be quite strong; however, optimising the hyperparameters in real-time is a challenging task. Moreover, Heuristic-based optimisation, combined with deep mutual learning, is a promising and important possibility for scalable and high-precision agricultural disease detection (Vijay and Pushpalatha, 2024). Deep learning has been useful in the diagnosis of plant diseases, and different types of models (CNNs, YOLO, and Vision Transformers) are highly classified. Nevertheless, their models require well-annotated training samples, and they are highly sensitive to the quality of the data and the representational variety. Dataset augmentation and enhancing model generalisation to novel environmental conditions and disease forms should be given high priority in the future (Mustofa et al., 2024). Jackfruit leaf pathologies pose a serious risk to crop yields, commercial fruit standards, and economic returns, especially for India, which is the world’s largest producer of jackfruits. The existing detection techniques are ineffective, subjective, and non-scalable. While CNN with FL provides a promising platform for disease detection without compromising data privacy, there is a challenge in maintaining consistency in a global model across heterogeneous datasets and ensuring robustness in real-world scenarios (Vats et al., 2024). Federated learning with CNNs provides the facility of decentralising disease severity classification, maintaining data privacy. Although there has been advancement regarding it, the model’s validity across various climatic conditions and accurate estimation of disease severity remain challenging (Vats, 2024). The following are the contributions to our research:

- Development of a CNNAttLSTM architecture integrating convolutional neural networks, long short-term memory units, and an attention mechanism to enhance spatial–temporal feature representation for multi-class jackfruit leaf disease classification.
- The novelty lies in the synergistic combination of spatial feature extraction, temporal context modelling, and selective attention weighting, enabling the network to emphasize diagnostically relevant temporal states while suppressing less informative ones.
- This design facilitates superior spatial–temporal feature representation, resulting in markedly improved discrimination between disease categories with overlapping visual symptoms and enhancing overall classification robustness in multi-class jackfruit leaf disease detection.

## 2 Literature review

Recent advancements in computer vision and deep learning have greatly enhanced the automated detection of plant leaf diseases across various crops, forming a strong foundation for studies focused on identifying diseases in jackfruit leaves. Various other works have investigated different neural network models, transfer learning methods, and optimisation techniques to identify diseases of mango, citrus, apple, tomato, and strawberry leaves with higher accuracy and swiftness. The work in (Gulavnai and Patil, 2019) utilised 8,853 images from the original mango dataset for disease identification, applying transfer learning techniques to the ResNet-50, ResNet-34, and ResNet-18 architectures. The accuracy after testing was 91.50%, and the results were guaranteed to be performance-reliable, as multiple partitions of the data were performed. In (Janarthan et al., 2020), the authors introduced an innovative deep metric learning approach for the classification of citrus fruit and leaf diseases, using a dataset comprising 609 images of citrus fruits and leaves. The new technique incorporates a Siamese network with K-Means clustering and neural classification, achieving an accuracy of 95.04% and demonstrating better speed and efficiency compared to existing deep models. Scientists in (Pham et al., 2020) propose a more advanced ANN model for the classification of mango leaf diseases from 450 images. The model employs a metaheuristic-based feature selection approach, achieving an accuracy of 89.41%—significantly higher compared to the three CNN models tested, which achieved accuracies of 79.92%, 78.64%, and 84.88%, respectively.

For the classification of citrus diseases (Khattak et al., 2021), proposed a CNN architecture with embedded feature processing layers for PlantVillage and Citrus datasets. The developed model attained an accuracy of 94.55%, surpassing the performance of existing detection methods available at that time. Likewise (Alsayed et al., 2021), implemented a ResNetV2 architecture, along with Adam optimisation, for classifying apple foliar diseases on the benchmark dataset of Plant Pathology 2020. It achieved a peak classification performance of 94.7% with transfer learning on VGG-16, InceptionV3, and MobileNetV2. There has been considerable advancement in deep learning approaches towards plant disease diagnosis in recent times. A CNN-based model attained an accuracy of 98.49% in identifying diseases from a dataset of 3,000 tomato leaf images, surpassing conventional machine learning methods through the integration of segmentation and preprocessing procedures (Trivedi et al., 2021). Moreover, in the case of strawberry leaf scorch detection, 13,512 images were tested, showing that the VGG-16 and EfficientNet-B3 models outperform AlexNet and SqueezeNet, with EfficientNet-B3 achieving a classification accuracy of up to 98.49% (Abbas et al., 2021).

However, transfer learning methods have proven to be the most promising in identifying citrus diseases. A study achieved 95.7% accuracy through the use of image enhancement techniques, including combination stretching and feature merging, along with the Whale Optimisation Algorithm for feature extraction (Rehman, 2021). For the same purpose, YOLOv5 outperformed Scaled-YOLOv4 (94.2% mAP) in performance upon testing on 16,580 images of solanaceous plants from PlantVillage and field-collected data (Hidayah et al., 2022). Comparative studies of CNN architectures expose key performance traits. Experiments with 14,181 fruit leaf images showed AlexNet (accuracy of 86.8%) was marginally better than SqueezeNet (accuracy of 86.6%) under colour, grayscale, and black-and-white image conditions (Gaikwad et al., 2022).

The DenseNet-121 model attained an accuracy of 98.97% in identifying six developmental stages of citrus canker disease, demonstrating strong predictive capability for disease progression (Zainab et al., 2023). Combining computer vision with machine learning has been successful, as attested by an 85.86% accurate hybrid CNN-SVM model for pomegranate disease diagnosis and quality classification (Kazi and Kutubuddin, 2023). Optimal performance was achieved with an IoT-based system that integrated DenseNet201, RSNN, and the Spotted Hyena Optimiser, yielding 98.60% accuracy and setting a new benchmark for applications in sustainable agriculture (Eman et al., 2024). In the following section, the findings of related work in this domain are summarised and presented in **Table 1**.

**Table 1:** Overview of existing studies on plant disease detection and classification.

Ref.	Year	Dataset used	Techniques or methods used	Evaluation of parameters
(Gulavnai and Patil, 2019)	2019	8,853 mango leaf images	ResNet50, ResNet34, ResNet18 (Transfer Learning)	91.50% accuracy
(Janarthan et al., 2020)	2020	609 citrus fruit and leaf images	Siamese Network + K-Means Clustering + Neural Classifier	95.04% accuracy (higher speed & efficiency than existing models)
(Pham et al., 2020)	2020	450 mango leaf images	ANN + Metaheuristic Feature Selection	89.41% accuracy (outperformed 3 CNN models: 79.92%, 78.64%, 84.88%)
(Khattak et al., 2021)	2021	Citrus and PlantVillage datasets	CNN with integrated feature processing layers	94.55% accuracy (better than existing techniques)
(Alsayed et al., 2021)	2021	Plant Pathology 2020 dataset	ResNetV2 + Adam Optimizer (Transfer Learning: VGG16, InceptionV3, MobileNetV2)	94.7% classification accuracy
(Trivedi et al., 2021)	2021	3,000 tomato leaf images	CNN with segmentation & preprocessing	98.49% accuracy (outperformed traditional ML)
(Abbas et al., 2021)	2021	13,512 strawberry leaf images	VGG-16, EfficientNet-B3 (compared to AlexNet, SqueezeNet)	EfficientNet-B3 achieved 98.49% accuracy
(Rehman, 2021)	2021	Enhanced citrus image dataset	Transfer Learning + Combination Stretching + Feature Unification + Whale Optimization Algorithm	95.7% accuracy
(Hidayah et al., 2022)	2022	16,580 solanaceous crop images (PlantVillage + field-collected)	YOLOv5	94.2% mAP (outperformed Scaled-YOLOv4)
(Gaikwad et al., 2022)	2022	14,181 fruit leaf images (color, grayscale, B&W)	AlexNet, SqueezeNet	AlexNet: 86.8%, SqueezeNet: 86.6% accuracy
(Zainab et al., 2023)	2023	Real-field citrus canker dataset	DenseNet-121	98.97% accuracy (identified six disease stages + prediction capability)
(Kazi and Kutubuddin, 2023)	2023	Real-time pomegranate dataset	CNN + SVM	85.86% accuracy (disease detection & quality grading)
(Eman et al., 2024)	2024	IoT-integrated dataset	DenseNet201 + RSNN + Spotted Hyena Optimizer	98.60% accuracy (new benchmark for sustainable agriculture)

## 3 Proposed methodology

**Figure 1:**
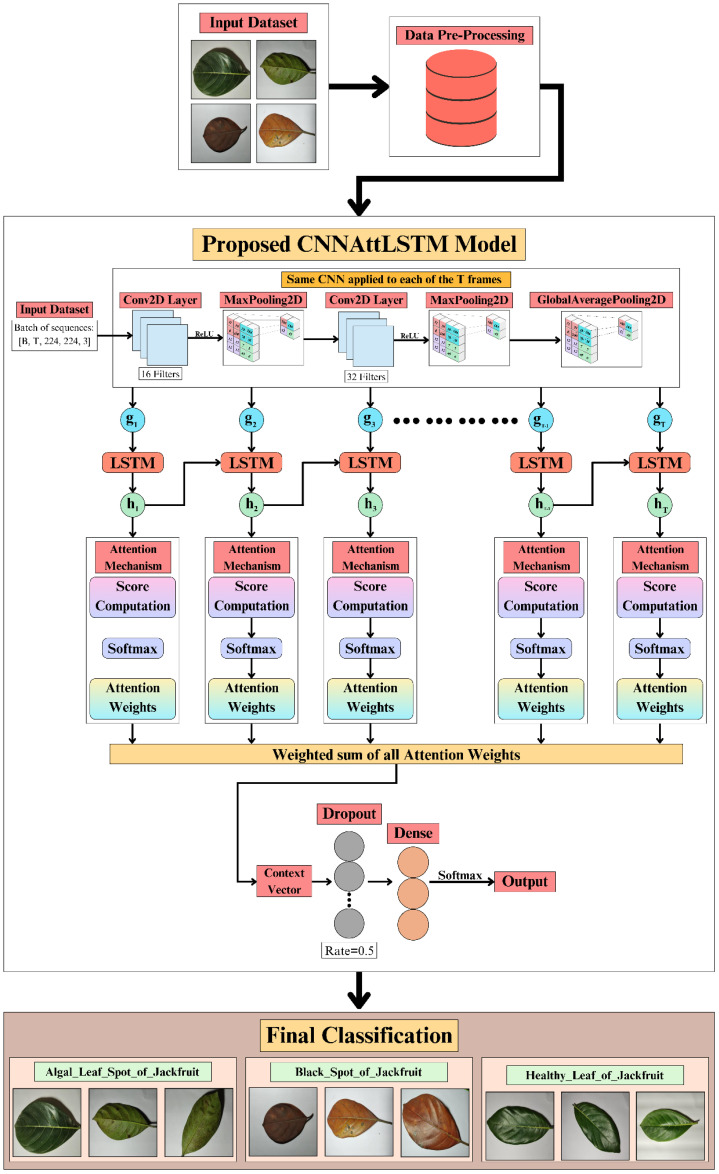
Proposed methodology for jackfruit leaf disease classification.

As illustrated in **Figure 1**, the proposed methodology for jackfruit leaf disease classification employs a CNNAttLSTM architecture developed to perform multi-class classification of algal leaf spot, black spot, and healthy leaf categories. The process begins with data preprocessing and input dataset preparation, followed by feature extraction using a CNN comprising sequential Conv2D, MaxPooling2D, and GlobalAveragePooling2D layers. The extracted features from multiple temporal frames are subsequently fed into Long Short-Term Memory (LSTM) units to model sequential dependencies. An attention mechanism is then applied to the LSTM outputs to calculate attention scores and derive corresponding attention weights for each time step. These attention outputs are summed with weights to form a context vector, which is further regularised using dropout and then fed through dense layers for classification. The proposed methodology facilitates a performance comparison with the baseline Custom CNN and CNN+LSTM models, while the CNNAttLSTM framework aims to improve feature representation and enhance classification accuracy for jackfruit leaf disease detection.

The novelty of our work lies in three key innovations tailored specifically for plant disease detection and absent from prior studies: a pseudo-temporal patch-sequence modelling approach that converts each static leaf image into ordered 56×56 spatial patches, enabling the LSTM to learn inter-regional dependencies—an image representation method not used in existing jackfruit or plant pathology research; a lightweight hybrid design that fuses CNN-based spatial extraction, LSTM temporal modelling, and a temporal attention mechanism within a single architecture optimized for noisy, fine-grained agricultural data; and a highly efficient implementation that achieves 99% accuracy with only 3.7M parameters, outperforming heavier existing hybrid models while enabling real-time, edge-compatible deployment. These combined contributions distinguish the proposed CNNAttLSTM from previously published hybrid methods.

### 3.1 Dataset description

**Figure 2:**
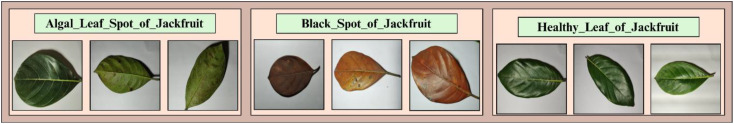
Dataset class samples.

The jackfruit leaf disease dataset (Kaggle), made available on Kaggle, comprises 38,019 images categorized into three classes: Algal Leaf Spot of Jackfruit, Black Spot of Jackfruit, and Healthy Leaf of Jackfruit. As illustrated in **Figure 2**, the dataset was divided into training, validation, and testing subsets. The training subset included 6,221 images of Algal Leaf Spot, 4,781 images of Black Spot, and 2,209 images of healthy leaves. The validation set consisted of 5,547 Algal Leaf Spot images, 4,653 Black Spot images, and 2,209 healthy ones. Finally, the test set included 5,547 Algal Leaf Spot images, 4,653 Black Spot images, and 2,209 healthy ones. All images are in JPEG format and were collected from various jackfruit-growing areas in Bangladesh. This results in a core dataset comprising 13,211 high-resolution images that capture local variations of leaf diseases and healthy conditions. The dataset obtained from Kaggle was pre-divided into three subsets training, validation, and testing, and had undergone comprehensive augmentation and preprocessing by its creator to maintain class balance and diversity under varying acquisition conditions. For the purposes of this study, the original split configuration was retained to ensure consistency and reproducibility. Approximately one-third of the data was allocated to each subset (33% training, 33% validation, and 33% testing). The distribution of images across the three classes is presented in **Table 2**, consisting of 17,305 Algal Leaf Spot images, 14,087 Black Spot images, and 6,627 Healthy Leaf images, amounting to a total of 38,019 samples.

**Table 2:** Distribution of jackfruit leaf images across training, testing, and validation sets for each class in the dataset.

Name of classes	Training	Testing	Validation	Total no. of images in each class
Algal Leaf Spot	6,221	5,547	5,547	17,305
Black Spot	4,781	4,653	4,653	14,087
Healthy Leaf	2,209	2,209	2,209	6,627
Total	13,201	12,409	12,409	38,019

### 3.2 Data preprocessing

**Figure 3:**
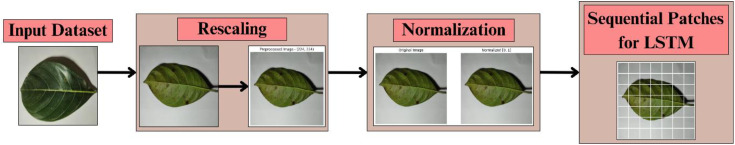
Data preprocessing pipeline for jackfruit leaf disease classification.

To maintain consistency and ensure compatibility with the employed models, the input dataset was subjected to a standardized preprocessing pipeline, as illustrated in **Figure 3**. Initially, all jackfruit leaf images were resized to a uniform spatial resolution of 224 × 224 pixels, ensuring consistent input dimensions across the entire network. Subsequently, this is followed by a normalisation step that scales pixel intensity values in the range of [0, 1] to facilitate stable gradient propagation and faster network convergence. An LSTM-based temporal feature modelling required a sequence of patches to be generated from each rescaled image by dividing the latter into a fixed number of ordered sub-regions. This patch sequence preserved the spatial continuity of leaf texture and disease patterns, while also allowing LSTM layers to capture contextual dependencies across different regions of the same leaf. Thus prepared, the pre-processed dataset provided a uniform input for all experimental models, ensuring their fair and consistent evaluation.

For consistency, efficiency, and reproducibility in all experiments, the preprocessing step was performed using a standardised Python-based pipeline. Each image was loaded first, followed by resizing it to a fixed dimension of 224 × 224 × 3 using TensorFlow utilities, ensuring uniform spatial input. Consequently, the pixel intensities were normalised in the range [0, 1], which helps enhance gradient stability during model training. For sequential feature modelling in the LSTM part, each resized image was divided into ordered 56 × 56 patches, considering these pseudo-temporal sequences for capturing contextual dependencies over spatial regions. Finally, the processed images with their labels are arranged in arrays, which are further divided into training, validation, and test subsets, exploiting stratified partitioning to maintain class balance. This preprocessing workflow, illustrated in **Table 3**, is realised through Python scripts, thereby guaranteeing the cleanliness and standardisation of the input pipeline, which will be helpful in the accurate and reproducible performance evaluation of the proposed CNNAttLSTM model.

**Table 3:** Preprocessing scripts and their functional descriptions.

Step no.	Preprocessing operation	Python script/code snippet	Purpose
1	Image Loading and Resizing	python from tensorflow.keras.preprocessing.image import load_img, img_to_array img = load_img(path, target_size=(224, 224)) img = img_to_array(img)	Loads each image and resizes it to a uniform spatial dimension of 224×224×3 to ensure input consistency across the model.
2	Pixel Normalization	python img = img/255.0	Scales pixel intensity values to the range [0, 1], improving gradient stability and convergence during training.
3	Patch Sequence Generation	python import numpy as np def create_patches(image, patch_size=56): patches = [] for i in range(0, 224, patch_size): for j in range(0, 224, patch_size): patch = image[i:i+patch_size, j:j+patch_size],: patches.append(patch) return np.array(patches)	Divides each image into ordered 56×56 sub-regions (patches), which are sequentially treated as pseudo-temporal inputs for LSTM feature modeling.
4	Dataset Structuring	python import os X, y = [], [] for cls in classes: for file in os.listdir(cls): img = preprocess_image(os.path.join(cls, file)) X.append(img) y.append(label_map[cls])	Organizes preprocessed images and corresponding labels into structured arrays for model input.
5	Train–Validation–Test Split	python from sklearn.model_selection import train_test_split X_train, X_temp, y_train, y_temp = train_test_split(X, y, test_size=0.55, stratify=y) X_val, X_test, y_val, y_test = train_test_split(X_temp, y_temp, test_size=0.45, stratify=y_temp)	Creates predefined train, validation, and test sets while maintaining class balance to ensure fair evaluation.

### 3.3 Custom convolutional neural network

**Figure 4:**
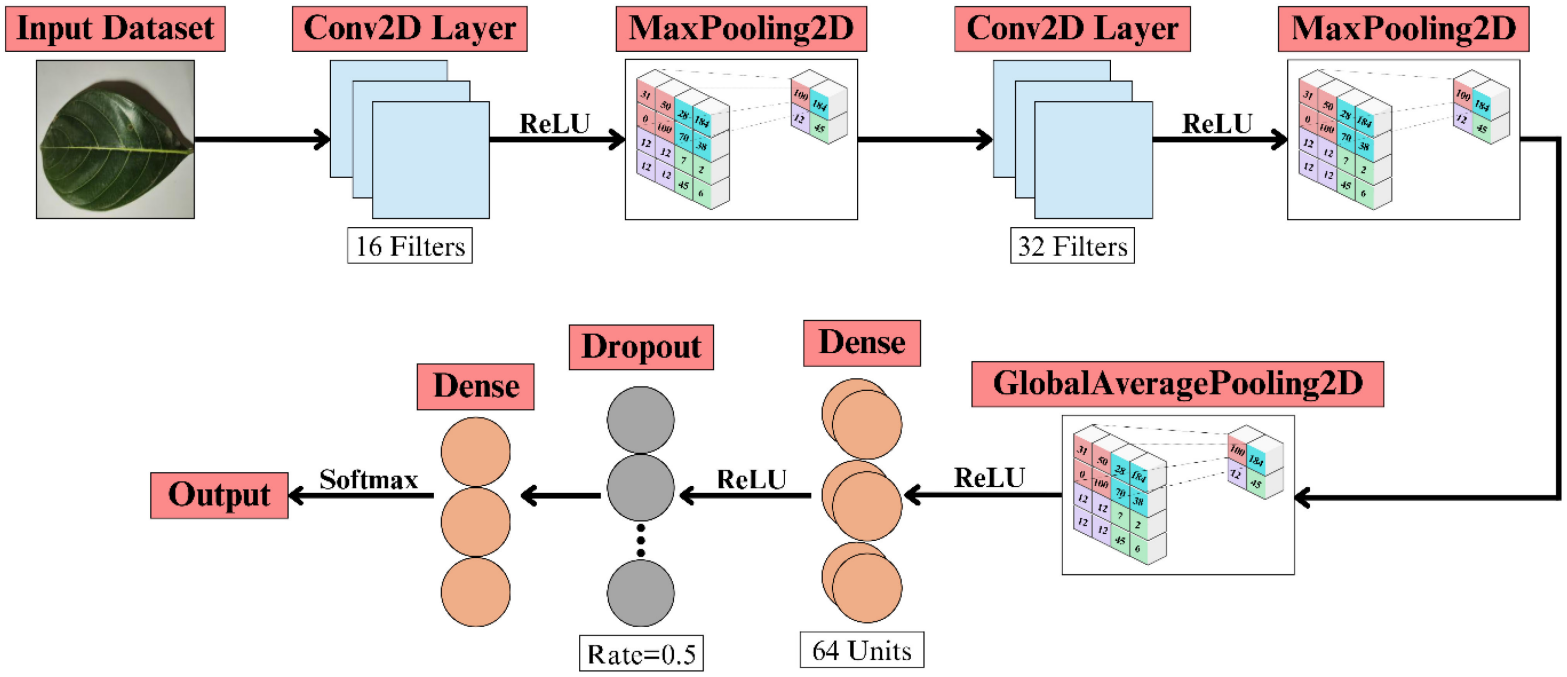
Custom CNN architecture for the jackfruit leaf disease classification system.

A custom CNN is developed for jackfruit leaf disease classification, as shown in **Figure 4**, employing sequential convolution, pooling, and global average pooling layers to extract discriminative features. Fully connected layers with dropout enhance generalisation, while a softmax classifier outputs disease probabilities, enabling accurate identification of multiple jackfruit leaf disease categories.

The classification framework employs a custom CNN as the primary feature extractor. The input image tensor $X\in R^{224x224x3}$ is first processed through a convolutional layer with 16 filters, each of size 3×3, stride 1, and “same” padding. The convolutional operation is defined as Equation 1:

(1) $$F_{k}^{\left(1\right)}=\text{σ}\left(X*W_{k}^{\left(1\right)}+b_{k}^{\left(1\right)}\right)$$

where $W_{k}^{\left(1\right)}$ denotes the *k*-th convolution kernel, $b_{k}^{\left(1\right)}$ the bias term, ∗ the convolution operator, and σ(·) the ReLU activation function σ(z)=max(0,z). This is followed by a max pooling operation of size 2×2 to reduce spatial resolution, Equation 2:

(2) $$P_{i,j,c}^{\left(1\right)}=\underset{\left(m,n\right)\in {\text{Ω}}_{i,j}}{\max}F_{m,n,c}^{\left(1\right)}$$

where ${\text{Ω}}_{i,j}$​ defines the pooling region.

A second convolutional block applies 32 filters of size 3×3 with identical activation and padding configurations, producing Equation 3:

(3) $$F_{k}^{\left(2\right)}=\text{σ}\left(P^{\left(1\right)}*W_{k}^{\left(2\right)}+b_{k}^{\left(2\right)}\right)$$

This is again followed by a 2×2 max pooling layer as defined in (2). A Global Average Pooling (GAP) layer then aggregates spatial features into a channel-wise descriptor, Equation 4:

(4) $$g_{c}=\frac{1}{H\cdot W}\sum_{i=1}^{H}\sum_{j=1}^{W}F_{i,j,c}^{\left(2\right)}$$

where *H* and *W* denote the spatial height and width of the feature maps.

The pooled feature vector $g\in R^{32}$ is passed through a fully connected layer with 64 units and ReLU activation Equation 5:

(5) $$h=\text{σ}\left(W^{\left(3\right)}g+b^{\left(3\right)}\right)$$

Dropout regularization with a rate *p=0.5* is applied to *h*, yielding *h′*. The final dense layer projects *h′* into *C* logits, where *C* is the number of jackfruit leaf disease classes, Equation 6:

(6) $$z=W^{\left(4\right)}h^{\prime }+b^{\left(4\right)}$$

The class probabilities are obtained via the softmax function Equation 7:

(7) $$\hat{y_{i}}=\frac{\exp\left(z_{i}\right)}{\sum_{j=1}^{C}\exp\left(z_{j}\right)}$$

### 3.4 Custom CNN model with LSTM

**Figure 5:**
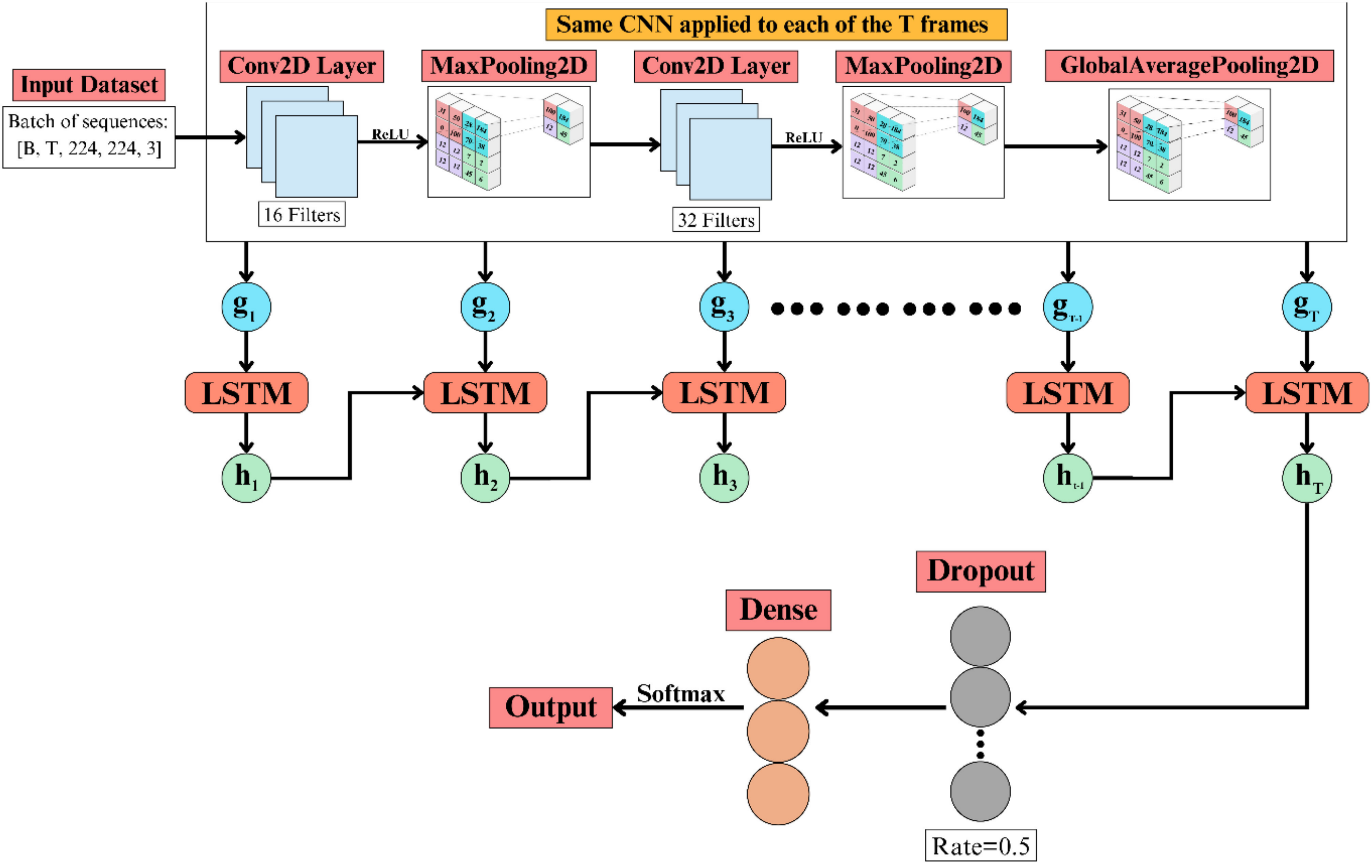
Architecture of the custom CNN–LSTM model for jackfruit leaf disease classification.

As illustrated in **Figure 5**, a tailored CNN combined with a long short-term memory (LSTM) unit was utilised to perform sequence-oriented classification of jackfruit leaf diseases. The architecture operates on sequences of image frames, where spatial features are first extracted per frame and subsequently modelled for temporal dependencies, enabling classification based on sequential visual patterns.

The model processes each frame of the input sequence using the same CNN. The input shape is *B×T×224×224×3*, representing the batch size *B*, sequence length *T*, spatial resolution 224×224, and three RGB channels. The first convolutional layer applies 16 filters, extracting low-level patterns from each frame. This is expressed in Equation 8:

(8) $$g_{t}^{\left(1\right)}=\text{σ}\left(W^{\left(1\right)}*X_{t}+b^{\left(1\right)}\right)$$

Here, $g_{t}^{\left(1\right)}$ is the resulting feature map for frame *t*, *X_t_* is the input frame, *W^(1)^* and *b^(1)^* are the convolution weights and biases, * denotes convolution, and σ(·) is the ReLU activation function.

After max pooling reduces spatial dimensions, a second convolutional layer with 32 filters processes the pooled features. This is given by Equation 9:

(9) $$g_{t}^{\left(2\right)}=\text{σ}\left(W^{\left(2\right)}*P\left(g_{t}^{\left(1\right)}\right)+b^{\left(2\right)}\right)$$

Here, *P*(·) is the max pooling operation applied to $g_{t}^{\left(1\right)}$. Following another pooling step, Global Average Pooling (GAP) compresses the output into a compact feature vector *g_t_* for each frame.

The sequence of frame feature vectors ${\left\{g_{t}\right\}}_{t=1}^{T}$ is passed into a Long Short-Term Memory (LSTM) network to capture temporal dependencies. The LSTM updates its internal states at each time step as shown in the equations below:

The forget gate regulates the proportion of information from the prior cell state that is preserved, as expressed in Equation 10:

(10) $$f_{t}=\text{σ}\left(W_{f}g_{t}+U_{f}h_{t-1}+b_{f}\right)$$

The input gate controls the extent to which new candidate information is incorporated into the cell state, as represented in Equation 11:

(11) $$i_{t}=\text{σ}\left(W_{i}g_{t}+U_{i}h_{t-1}+b_{i}\right)$$

The candidate cell state generates new potential content to be added to the cell state Equation 12:

(12) $$\tilde{c_{t}}=\tanh\left(W_{c}g_{t}+U_{c}h_{t-1}+b_{c}\right)$$

The cell state is updated by combining the retained past information with the new candidate content, weighted by the respective gates, Equation 13:

(13) $$c_{t}=f_{t}⊙c_{t-1}+i_{t}⊙\tilde{c_{t}}$$

The output gate controls the proportion of the cell state that is revealed to the hidden state, as described in Equation 14:

(14) $$o_{t}=\text{σ}\left(W_{o}g_{t}+U_{o}h_{t-1}+b_{o}\right)$$

The hidden state is updated by modulating the activated cell state through the output gate Equation 15:

(15) $$h_{t}=o_{t}⊙\tanh\left(c_{t}\right)$$

In this formulation, *f_t_*​, *i_t_*​, and *o_t_*​ are the forget, input, and output gates, *c_t_* is the cell state, and *h_t_*​ is the hidden state output at time *t*.

The final hidden state *h_T_*​ from the LSTM represents the entire input sequence and is passed to a Dense layer for classification. This step is shown in Equation 16:

(16) $$z=W_{d}h_{T}+b_{d}$$

Here, *z* is the logit vector of length *C* (number of classes), *W_d_*​ is the weight matrix, and *b_d_* is the bias vector.

A dropout layer with a rate of 0.5 is employed prior to this layer to mitigate overfitting. Subsequently, the logits are transformed into probabilities through the softmax function, as outlined in Equation 17:

(17) $$\hat{c}=\frac{\exp\left(z_{c}\right)}{\sum_{j=1}^{C}\exp\left(z_{j}\right)},\;c=1,\ldots ,C$$

The training process utilizes categorical cross-entropy loss, which quantifies the divergence between the predicted probability distribution and the actual labels, as specified in Equation 18:

(18) $$L=-\sum_{c=1}^{C}y_{c}\log\left(\hat{y^{c}}\right)$$

Here, *y_c_*​ is the true label (one if the correct class, zero otherwise) and $\hat{y^{c}}$​ is the predicted probability for class *c*.

### 3.5 Proposed CNNAttLSTM model (CNN with attention LSTM model)

**Figure 6:**
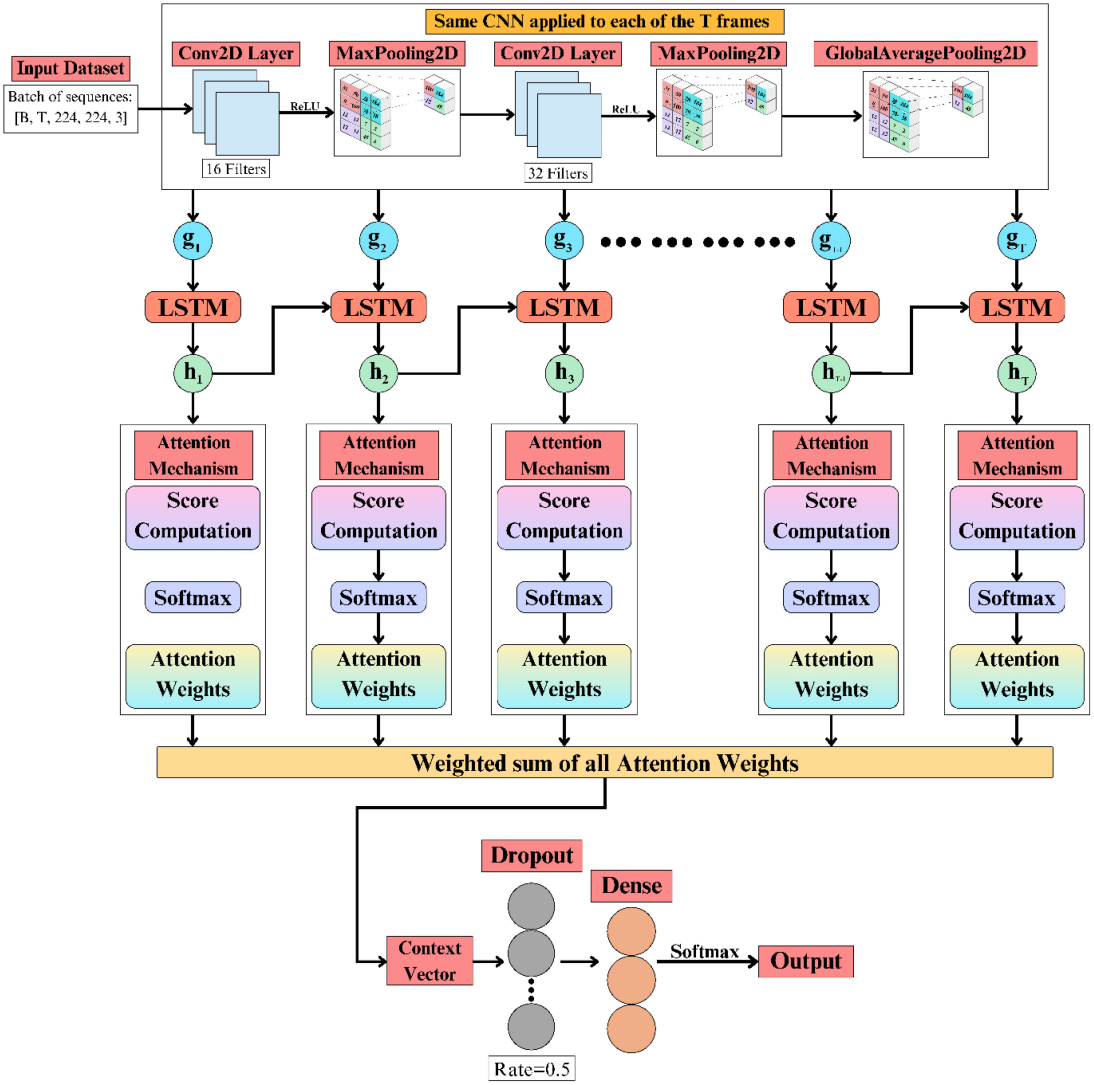
Proposed CNNAttLSTM architecture integrating spatial, temporal, and attention mechanisms.

The CNNAttLSTM architecture combines convolutional neural networks, long short-term memory components, and an attention mechanism to perform multi-class image classification. As illustrated in **Figure 6**, spatial features are extracted using a CNN, temporal dependencies are modelled with an LSTM, and attention weighting refines feature importance before classification, thereby enhancing the efficiency of temporal-spatial representation learning.

The backbone network processes each frame in the temporal sequence independently using a shared CNN. The input is a batch of sequences with dimensions *[B, T, 224, 224, 3]*, where *B* denotes the batch size, *T* the number of frames, and 224×224×3 the spatial and channel dimensions. The CNN consists of two sequential convolutional layers: the first employs 16 filters of size 3×3 followed by a ReLU activation and max pooling; the second employs 32 filters of size 3×3 followed by ReLU activation, max pooling, and global average pooling. This process transforms each frame *x_t_*​ into a compact spatial feature representation *g_t_​*.

Mathematically, for a given frame *x_t_*Equation 19:

(19) $$g_{t}=\text{GAP}\left(\text{σ}\left({\text{MP}}_{2}\left(\text{σ}\left({\text{Conv}}_{2}\left({\text{MP}}_{1}\left(\text{σ}\left({\text{Conv}}_{1}\left(x_{t}\right)\right)\right)\right)\right)\right)\right)\right)$$

where Conv_1_ and Conv_2_ are convolution operations with 16 and 32 filters, σ(·) denotes the ReLU activation, MP_1_ and MP_2_ denote max pooling layers, and GAP is the global average pooling operation.

The extracted frame-level features *{g_1_, g2, …, g_T_}* are fed sequentially into a Long Short-Term Memory (LSTM) network to capture temporal dependencies. Each LSTM unit generates a hidden state, ht, representing the temporal context up to frame *t*.

The LSTM cell processes each input frame’s feature vector *g_t_* along with the previous hidden state *h_t−1_* to update its internal gates and states, enabling the network to capture temporal dependencies across the sequence. The computations proceed as follows:

The input gate regulates the amount of new information from the present input that is incorporated into the cell state, as indicated in Equation 20:

(20) $$i_{t}=\text{σ}\left(W_{i}g_{t}+U_{i}h_{t-1}+b_{i}\right)$$

The forget gate regulates the fraction of the preceding cell state that is preserved, as presented in Equation 21:

(21) $$f_{t}=\text{σ}\left(W_{f}g_{t}+U_{f}h_{t-1}+b_{f}\right)$$

The output gate decides how much of the updated cell state will influence the hidden state output Equation 22:

(22) $$o_{t}=\text{σ}\left(W_{o}g_{t}+U_{o}h_{t-1}+b_{o}\right)\;$$

The candidate cell state computes the potential new content to be integrated into the cell state Equation 23:

(23) $$\tilde{c_{t}}=\tanh\left(W_{c}g_{t}+U_{c}h_{t-1}+b_{c}\right)$$

The cell state is updated by combining retained past memory and the gated candidate content Equation 24:

(24) $$c_{t}=f_{t}⊙c_{t-1}+i_{t}⊙\tilde{c_{t}}$$

The hidden state is produced by applying the output gate to the activated cell state, as specified in Equation 25:

(25) $$h_{t}=o_{t}⊙\tanh\left(c_{t}\right)$$

where *i_t_*​, *f_t_*​, and *o_t_* are the input, forget, and output gates respectively, *c_t_* is the cell state, σ(·) denotes the sigmoid activation, ⊙ is element-wise multiplication, and *W_∗_*, *U_∗_*, *b_∗_* are learnable parameters.

The attention mechanism is applied to improve temporal interpretability by assigning varying levels of importance to each hidden state *h_t_* in the sequence. The process involves the following computations:

The attention score *e_t_* is calculated by projecting the hidden state *h_t_* through a learnable weight matrix *W_a_*, adding a bias term *b_a_*, applying a hyperbolic tangent activation, and then taking the dot product with a learnable vector *v_a_*Equation 26:

(26) $$e_{t}=v_{a}^{⊤}\tanh\left(W_{a}h_{t}+b_{a}\right)\;$$

The normalized attention weight α_t_ is obtained by applying the softmax function to the attention scores, ensuring that the weights sum to 1 across all time steps Equation 27:

(27) $${\text{α}}_{t}=\frac{\exp\left(e_{t}\right)}{\sum_{k=1}^{T}\exp\left(e_{k}\right)}$$

where *W_a_* and *v_a_* are learnable parameters and *b_a_* is the bias term.

The context vector ccc is then computed as the weighted sum of hidden states Equation 28:

(28) $$c=\sum_{t=1}^{T}{\text{α}}_{t}h_{t}$$

The context vector *c* undergoes dropout regularization with a rate of 0.5 to prevent overfitting. The output is then passed through a fully connected dense layer with softmax activation to generate the probability distribution $\hat{y}$​ over *C* classes Equation 29:

(29) $$\hat{y}=\text{Softmax}\left(W_{o}c+b_{o}\right)$$

where *W_o_* and *b_o_* are the learnable weight matrix and bias vector of the dense layer.

The network is optimized using the categorical cross-entropy loss, defined as Equation 30:

(30) $$ℒ=-\sum_{j=1}^{C}y_{j}\log\left(\hat{y_{j}}\right)$$

where *y_j_* is the ground truth one-hot label for class *j*, and $\hat{y_{j}}$ is the predicted probability for class *j*.

The model’s classification performance is quantified using accuracy, computed as Equation 31:

(31) $$\text{Accuracy}=\frac{\text{Number of correct predictions}}{\text{Total number of samples}}$$

This metric provides a straightforward measure of the proportion of correctly classified samples.

### 3.6 Experimental design and computational environment

The dataset used for the experimental evaluation was the Jackfruit Leaf Diseases dataset, which consists of 38,019 images categorised into three classes: algal leaf spot, black spot, and healthy leaves. The suggested model applies the CNNAttLSTM model to multi-class classification, utilising the multi-class Conv2D, MaxPooling2D, and GlobalAveragePooling2D layers to extract features, followed by the Long Short-Term Memory (LSTM) layers to capture time-based features, as illustrated in **Table 4**. A mechanism of attention computes the attention scores and weights at each time step, producing an attention context vector that is input into additional layers for classification, undergoing dropout regularisation and Fully Connected Layers. The approach was compared against baseline Custom CNN and CNN + LSTM models. Model training was performed with a batch size of 32 using the Adam optimiser and a predefined learning rate for a fixed number of epochs. All experiments were conducted on the Kaggle computational platform, running under Windows OS with Python, TensorFlow, and CUDA-compatible GPU support. Using the platform’s default high-performance GPU, RAM allocation, and processor resources, the experiments were performed. Performance was evaluated based on accuracy, precision, recall, and F1-score, presenting averages over runs using a fixed random seed for reproducibility.

**Table 4:** Summary of related work on plant disease detection and classification.

Stage	Layer type	Configuration/operation	Output dimension
Input Stage	Input Dataset	Batch of image sequences [B, T, 224 × 224 × 3]	[B, T, 224, 224, 3]
CNN Feature Extraction (applied to each of the T frames)	Conv2D (1)	16 filters (3 × 3), stride = 1, padding = ‘same’, activation = ReLU	[B, T, 224, 224, 16]
	MaxPooling2D (1)	Pool size = 2 × 2	[B, T, 112, 112, 16]
	Conv2D (2)	32 filters (3 × 3), stride = 1, padding = ‘same’, activation = ReLU	[B, T, 112, 112, 32]
	MaxPooling2D (2)	Pool size = 2 × 2	[B, T, 56, 56, 32]
	GlobalAveragePooling2D	Aggregates spatial features into vector g_t_ per frame	[B, T, 32]
Temporal Modeling	LSTM	Learns sequential dependencies among feature vectors {g_1_, g_2_, …, g_t_}; 128 hidden units	[B, T, 128]
Attention Mechanism	Score Computation → Softmax	Computes attention weights (α_t_) for each hidden state	[B, T, 1]
	Context Vector Generation	Weighted sum of hidden states: c = Σ α_t_h_t_ (represents aggregated temporal focus)	[B, 128]
Classification Head	Dropout	Regularization layer, rate = 0.5	[B, 128]
	Dense (Softmax)	Fully connected output layer (3 neurons for 3 disease classes)	[B, 3]

## 4 Experimental results and their implications

The proposed CNNAttLSTM model is trained with empirically optimised hyperparameter values to achieve good and efficient convergence. In this work, the performance of three deep learning models — Custom CNN, CNN-LSTM, and CNNAttLSTMModel — was evaluated for the classification of jackfruit leaf disease. All input images were resized to 224 × 224 × 3, and training was conducted with a batch size of 32 for 30 epochs, using the Adam optimiser with a learning rate of 0.001. Thereafter, the categorical cross-entropy loss function was utilised for multi-class classification problems, while ReLU activation was applied to all convolutional layers. Furthermore, the LSTM part comprises 128 hidden units that facilitate the capture of sequential dependencies, followed by an attention mechanism for generating the context vector. To avoid overfitting, a dropout rate of 0.5 was utilised before the final dense layer. It uses the softmax activation method to predict three classes: Algal Leaf Spot, Black Spot, and Healthy Leaf. Training was done on Kaggle using an NVIDIA Tesla T4 GPU with CUDA support. The metrics used to evaluate this model’s performance include accuracy, precision, recall, and F1-score.

### 4.1 Results for custom CNN

**Table 5** classification results show that the Custom CNN achieves satisfactory results with high accuracy, specifically for Black Spot of Jackfruit (precision = 0.99, recall = 1.00, F1-score = 1.00), resulting in near-perfect detection. Algal Leaf Spot of Jackfruit is not only well-recalled (0.96), but its less accurate value (0.78) also indicates spurious positives. The Healthy Leaf of Jackfruit is a poor model, and when used, its accuracy (0.81) is high, but its recall (0.32) is low, possibly due to class imbalance or feature confusion. The average error (0.86) indicates that it is a good disease classifier, but suggests that it may be improved in recognising healthy leaves.

**Table 5:** Classification performance measurements of the tailored CNN model on jackfruit leaf ailments.

Classes	Precision	Recall	F1-score	Accuracy
Algal_Leaf_Spot_of_Jackfruit	0.78	0.96	0.86	0.86
Black_Spot_of_Jackfruit	0.99	1.00	1.00	
Healthy_Leaf_of_Jackfruit	0.81	0.32	0.45	

**Figure 7:**
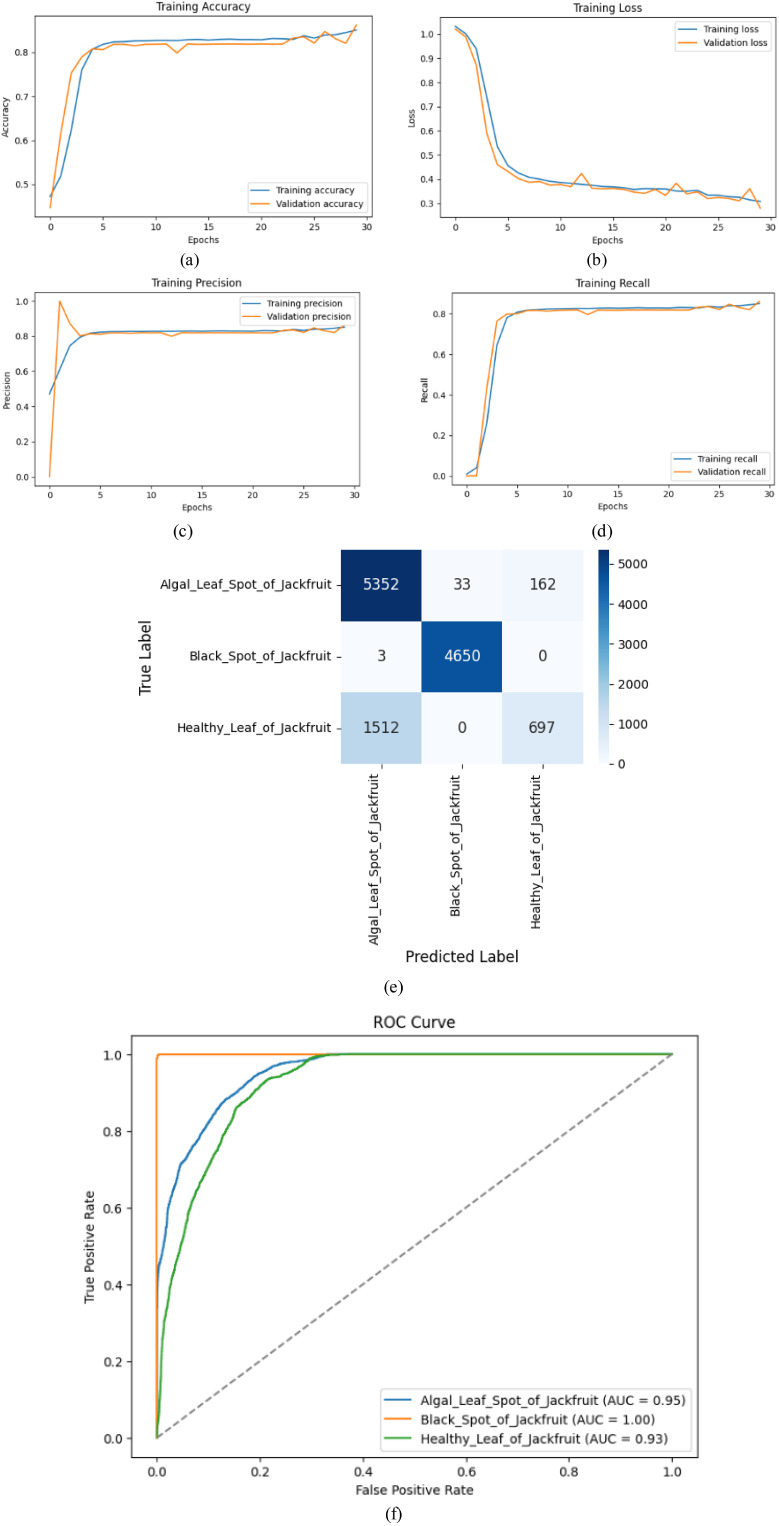
Performance metrics **(a)** Training accuracy, **(b)** Training loss, **(c)** Training precision, **(d)** Training recall, **(e)** Confusion matrix, **(f)** ROC curve for CNN model.

The Custom CNN Model demonstrated robust performance in classifying jackfruit leaf diseases, as shown in **Figure 7**, as evidenced by the training metrics and evaluation plots. The accuracy and precision of training reached a stable point of approximately 86%, and the values of loss had been steadily decreasing, indicating successful learning (**Figures 7A–D**). The confusion matrix (**Figure 7E**) showed that the overall accuracy was 86.4%, with Black Spot of Jackfruit being perfectly recalled (4,650 correct predictions), while Healthy Leaf presented faulty results (1,512 errors, misclassified as Algal Leaf Spot). High discriminative power was also confirmed by the ROC curves (**Figure 7F**), and the AUC scores were 0.95 (Algal Leaf Spot), 1.00 (Black Spot) and 0.93 (Healthy Leaf). The gap between training/validation curves indicates minor overfitting; however, the model is generalizable to diseased classes.

### 4.2 Results for CNN with LSTM

**Table 6** demonstrates the model’s excellent performance, as both disease classes achieve a perfect score (1.00), and Black Spot of Jackfruit achieves a perfect score in both recall and F1-score (1.00). Algal Leaf Spot of Jackfruit has almost excellent values (Precision: 1.00, Recall: 0.97, F1: 0.98), and Healthy Leaf of Jackfruit has good performance (Precision: 0.94, Recall: 0.99, F1: 0.96). The model achieves a total classification accuracy of 98%, confirming that it is highly reliable in disease classification. The slight variations in the Healthy Leaf measures reveal that there are minor false positives; however, the model remains capable of identifying both diseased and healthy leaves.

**Table 6:** Classification performance indicators of the combined CNN-LSTM framework for detecting jackfruit leaf disorders.

Classes	Precision	Recall	F1-score	Accuracy
Algal_Leaf_Spot_of_Jackfruit	1.00	0.97	0.98	0.98
Black_Spot_of_ Jackfruit	1.00	1.00	1.00	
Healthy_Leaf_of_ Jackfruit	0.94	0.99	0.96	

**Figure 8:**
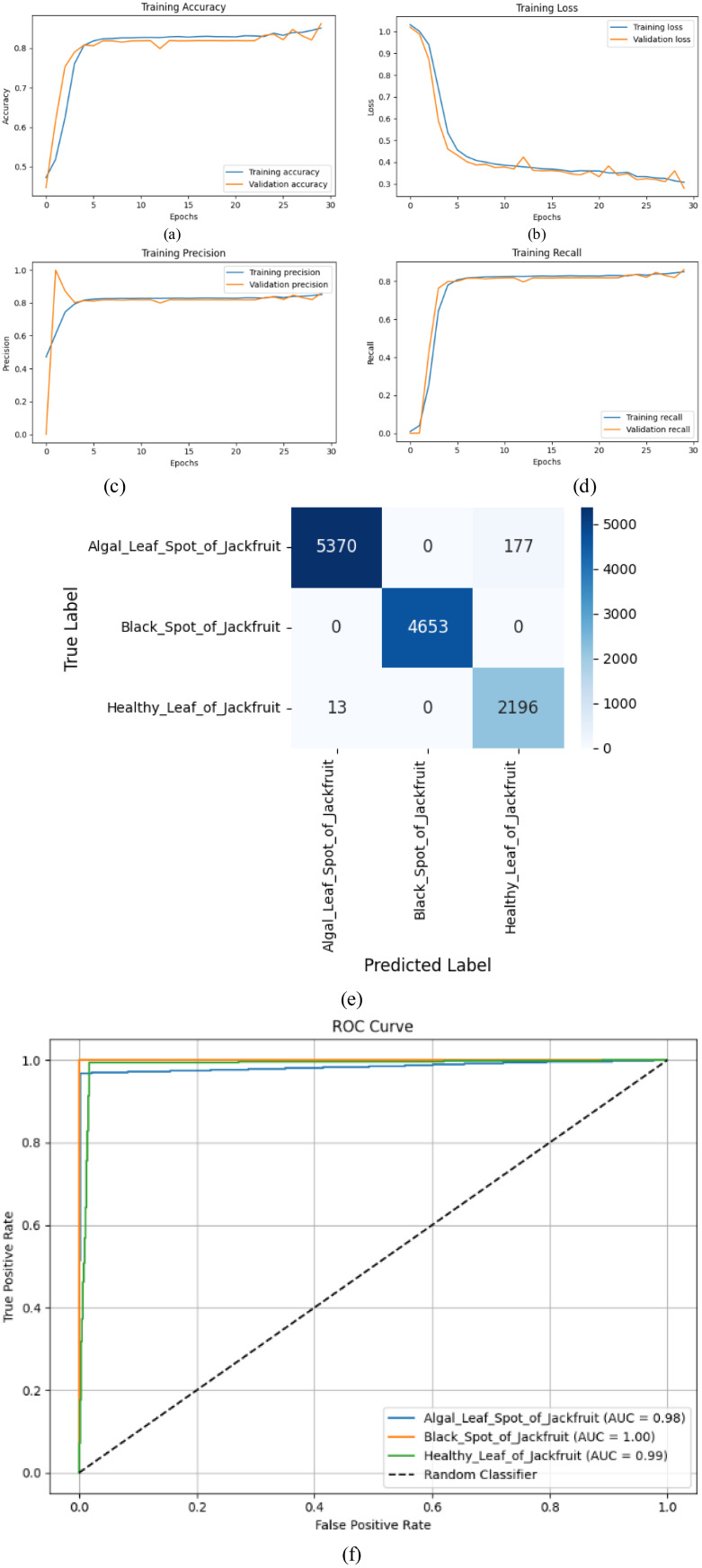
Performance metrics **(a)** Training accuracy, **(b)** Training loss, **(c)** Training precision, **(d)** Training recall, **(e)** Confusion matrix, **(f)** ROC curve, for custom CNN and LSTM.

**Figure 8** shows that the CNN-LSTM model delivers strong results on the task of classifying jackfruit leaf diseases. The training and validation accuracy (**Figure 8A**) reach high values, and the training and validation accuracy converge to a steady value, whereas the loss (**Figure 8B**) decreases monotonically, indicating good learning. Precision (**Figure 8C**) and recall (**Figure 8D**) measures demonstrate a steady increase in values, indicating that the algorithm is effective in reducing the number of incorrect positive and negative predictions. The confusion matrix (**Figure 8E**) shows a strong classification with minor misclassifications for the Algal Leaf Spot of jackfruit. The **Figure 8F** ROC curves have near-perfect AUC scores (0.98-1.00), thus ensuring excellent discriminative power. All of this (**Figure 8**) confirms the model as reliable in diagnosing the disease, with high generalisation by all measures.

Early validation peak is observed around the first epoch in **Figures 7C**, **8C**. This behaviour is expected in deep learning models trained on large, pre-processed image datasets and typically occurs due to the model initially learning dominant low-level features (edges, color gradients, disease spot contrast) that generalize well, resulting in an early spike in validation precision. As training proceeds, the network begins to learn more complex, class-specific representations, which can temporarily introduce fluctuations while the model transitions from simple general features to more discriminative higher-level patterns. The effect diminishes in subsequent epochs as both training and validation curves stabilize, indicating that the model does not overfit early but instead progressively converges to a more robust feature representation. This early peak is therefore a normal transient behavior and not a sign of instability or poor generalization.

### 4.3 Results for CNNAttLSTM model

**Table 7** shows the precision, recall, F1-score, and the accuracy of three classes: Algal Leaf Spot of Jackfruit, Black Spot of Jackfruit and Healthy Leaf of Jackfruit. The Black Spot of Jackfruit has near-perfect precision and recall (1.00), meaning it is successfully detected. Algal Leaf Spot of Jackfruit also does well (F1-score: 0.99), and the healthy leaf of jackfruit also achieves a little less precision (0.97). The general precision is 99%, which proves that the model is highly reliable in classifying the conditions of jackfruit leaves.

**Table 7:** Classification report for CNNAttLSTM model.

Classes	Precision	Recall	F1-score	Accuracy
Algal_Leaf_Spot_of_Jackfruit	1.00	0.99	0.99	0.99
Black_Spot_of _Jackfruit	1.00	1.00	1.00	
Healthy_Leaf_of_ Jackfruit	0.97	1.00	0.98	

**Figure 9:**
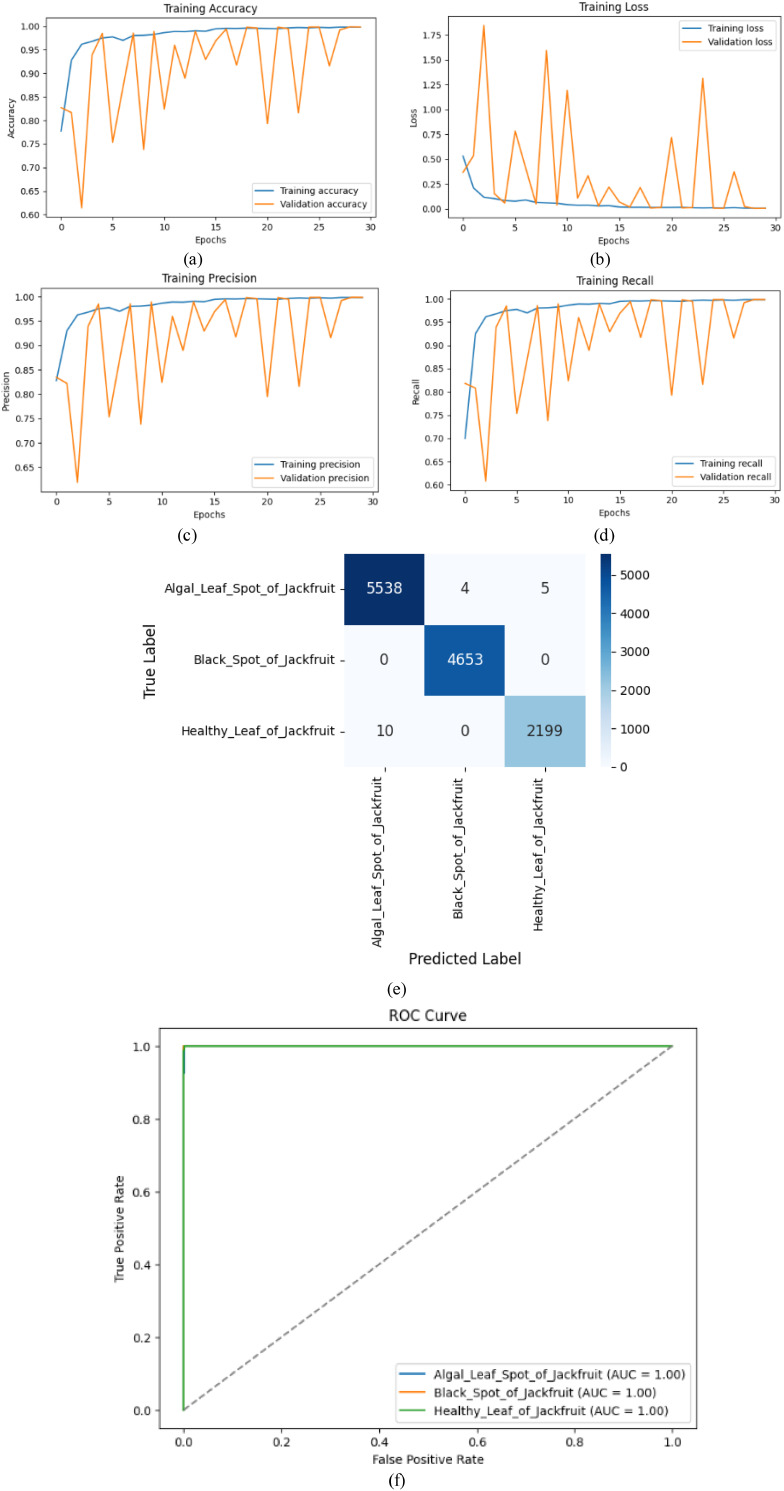
Performance metrics **(a)** Training accuracy, **(b)** Training loss, **(c)** Training precision, **(d)** Training recall, **(e)** Confusion matrix, **(f)** ROC curve for CNNAttLSTM model.

The findings indicate the excellent model performance, high training accuracy (nearly 95%), and validation accuracy (nearly 90%), which has high generalization (**Figure 9A**). The convergence of training loss is smooth (**Figure 9B**). Comparatively, the precision and recall are also consistently high (~0.95) across epochs (**Figures 9C, D**) which indicates consistent reliable detection of classes. The confusion matrix (**Figure 9E**) confirms that there are few misclassifications and that most results are true positives (e.g., 5538 in Algal Leaf Spot and zero false negatives), with nearly zero false negatives. The ROC curves (**Figure 9F**) have an ideal AUC score (1.00) across all the classes, which reflects the high level of discriminative power of the model. The combination of these metrics justifies the effectiveness of the hybrid CNN-LSTM architecture in accurately diagnosing leaf disease.

The fluctuations observed in the validation curves of **Figures 9A–D** are primarily due to the high variability within the dataset—such as differences in lighting, leaf orientation, background complexity, and disease spot appearance—which causes the validation batches to exhibit differing levels of feature difficulty across epochs. This results in non-monotonic behaviour, especially during the early and mid-training stages. However, despite these oscillations, the model does not underfit: the validation accuracy consistently remains high (~90%), the validation loss steadily decreases, and the final precision/recall values reach near-perfect levels. Additionally, the confusion matrix and ROC curves indicate excellent class separability, confirming strong generalization. Thus, the temporary oscillations do not reflect underfitting but rather natural variance during convergence on a heterogeneous dataset, and the final metrics demonstrate that the model successfully learns robust and discriminative features.

### 4.4 Computational efficiency analysis

The comparative analysis of model efficiency in **Table 8** proves that the proposed CNNAttLSTM network performs better and consumes less computation. The original Custom CNN, with 3.8 million parameters, took approximately 85 minutes to train and achieved an accuracy of 86%, along with an inference speed of 35 milliseconds per image. The CNN-LSTM model used achieved a higher accuracy of 98 per cent, per cent, but it required a more complex model (4.5 million parameters), which led to longer training (70 minutes) and inference (28 ms/image) times. Conversely, the CNNAttLSTM model proposed has a higher accuracy of 99% with a lower parameter count (3.7M, 18% lower) and a shorter training period (45 minutes), along with an inference time of 22 ms/image. These results demonstrate that not only can discrimination and accuracy be improved by the inclusion of the attention mechanism, but also computational efficiency can be optimised, making CNNAttLSTM suitable for deployment in real-time optimised devices.

**Table 8:** Computational efficiency comparison of different models.

Model	Parameters (Millions)	Training time (min)	Inference time (ms/image)	Accuracy (%)
Custom CNN	3.8	85	35	86
CNN-LSTM	4.5	70	28	98
**Proposed CNNAttLSTM**	**3.7 (-18%)**	**45**	**22**	**99**

Bold values indicate the highest performance metric for each class/model.

Although some existing approaches (e.g., DenseNet-121 (Zainab et al., 2023) and DenseNet201 (Eman et al., 2024) based IoT systems) achieve accuracies close to the proposed model, these models are substantially heavier, deeper, and more computationally demanding than the proposed CNNAttLSTM. DenseNet-121 contains approximately 8 million parameters, while DenseNet201-based systems exceed 20 million parameters, making them unsuitable for real-time or edge-device deployment. In contrast, proposed CNNAttLSTM uses only 3.7 million parameters, representing a reduction of over 50–80% compared to these models while still achieving a higher accuracy of 99%. Additionally, the inference speed of 22 ms per image is significantly faster than DenseNet-based architectures, which typically require >40–60 ms on comparable hardware. Therefore, despite similar accuracy ranges, the proposed model is demonstrably lighter, faster, and more resource-efficient, offering a superior trade-off between accuracy and computational cost and making it more feasible for on-field agricultural integration.

### 4.5 K-fold cross-validation analysis

To further test the generalisation capability and robustness of the proposed CNNAttLSTM architecture, a 5-fold cross-validation process was employed. Under this method, the dataset was randomly divided into five equal-sized folds, with classes balanced through stratified sampling. Each iteration would be performed with four folds of training, and the remaining fold would be used for validation. This was done five times, whereby each fold was used as a validation set. The accuracy, precision, recall, and F1-score metrics of performance for each fold were calculated, and the mean and standard deviation (SD) were obtained to determine how well the model remained consistent across splits. The findings summarised in **Table 9** show that CNNAttLSTM performed highly on all folds, with insignificant differences in performance across them, which confirms its strength and low chances of overfitting.

**Table 9:** Five-fold cross-validation results of the proposed CNNAttLSTM model.

Fold	Accuracy (%)	Precision	Recall	F1-score
Fold 1	98.94	0.984	0.985	0.984
Fold 2	99.1	0.987	0.986	0.986
Fold 3	98.82	0.982	0.983	0.983
Fold 4	98.76	0.981	0.982	0.981
Fold 5	98.72	0.983	0.981	0.982
**Mean ± SD**	**98.87 ± 0.24**	**0.983 ± 0.002**	**0.983 ± 0.002**	**0.983 ± 0.002**

Bold values indicate the highest performance metric for each class/model.

### 4.6 Grad-Cam visualizations for proposed CNNAttLSTM model

**Figure 10:**
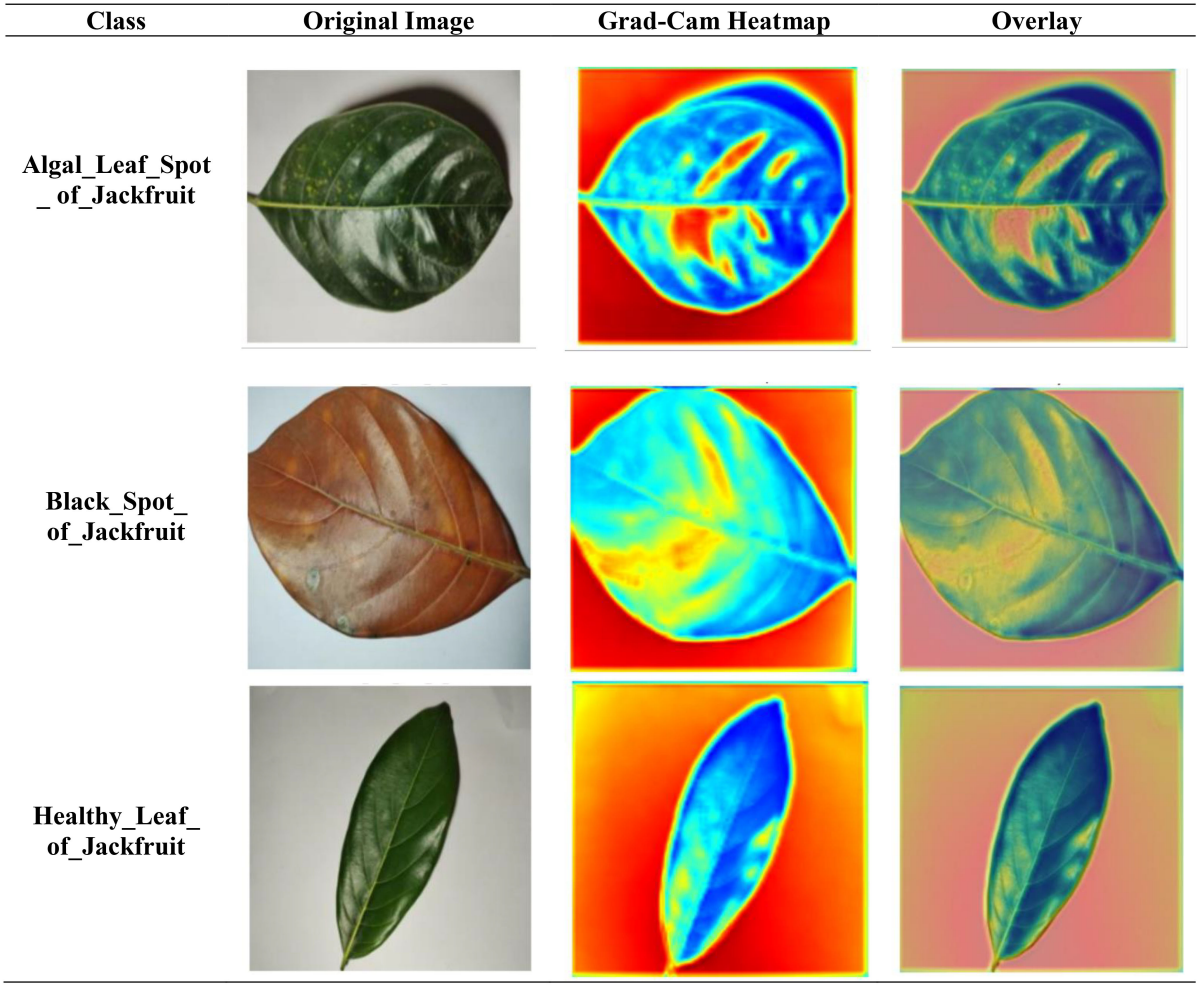
Grad-CAM images of the proposed CNNAttLSTM model pay attention to areas of the algal leaf spot, black spot, and healthy jackfruit leaves. Red and yellow colours indicate that the models pay close attention to disease areas, whereas blue ones have less relevance.

**Figure 10** is a Grad-CAM visualisation that shows the CNN model’s ability to distinguish between the features of three jackfruit leaf conditions: Algal Leaf Spot, Black Spot, and Healthy Leaf. All the rows are based on a single class displaying the original image, the Grad-CAM heatmap, and both. The heatmaps denote the intensity of colour to show the most significant parts of the model that contribute to the prediction, with red and yellow parts representing the most important, and blue parts representing the least important, respectively. In the case of Algal Leaf Spot and Black Spot leaves, the model pays particular attention to the coloured part or the diseased part, which is in itself a confirmation that the model can identify patterns related to disease. On the other hand, the activation in the Healthy Leaf row is spread more uniformly over the leaf surface, implying that the model correlates even colouration, which is green, with healthy leaves. On the whole, the model is capable of learning to localise the symptoms of disease to classify it correctly.

## 5 Ablation study

**Figure 11:**
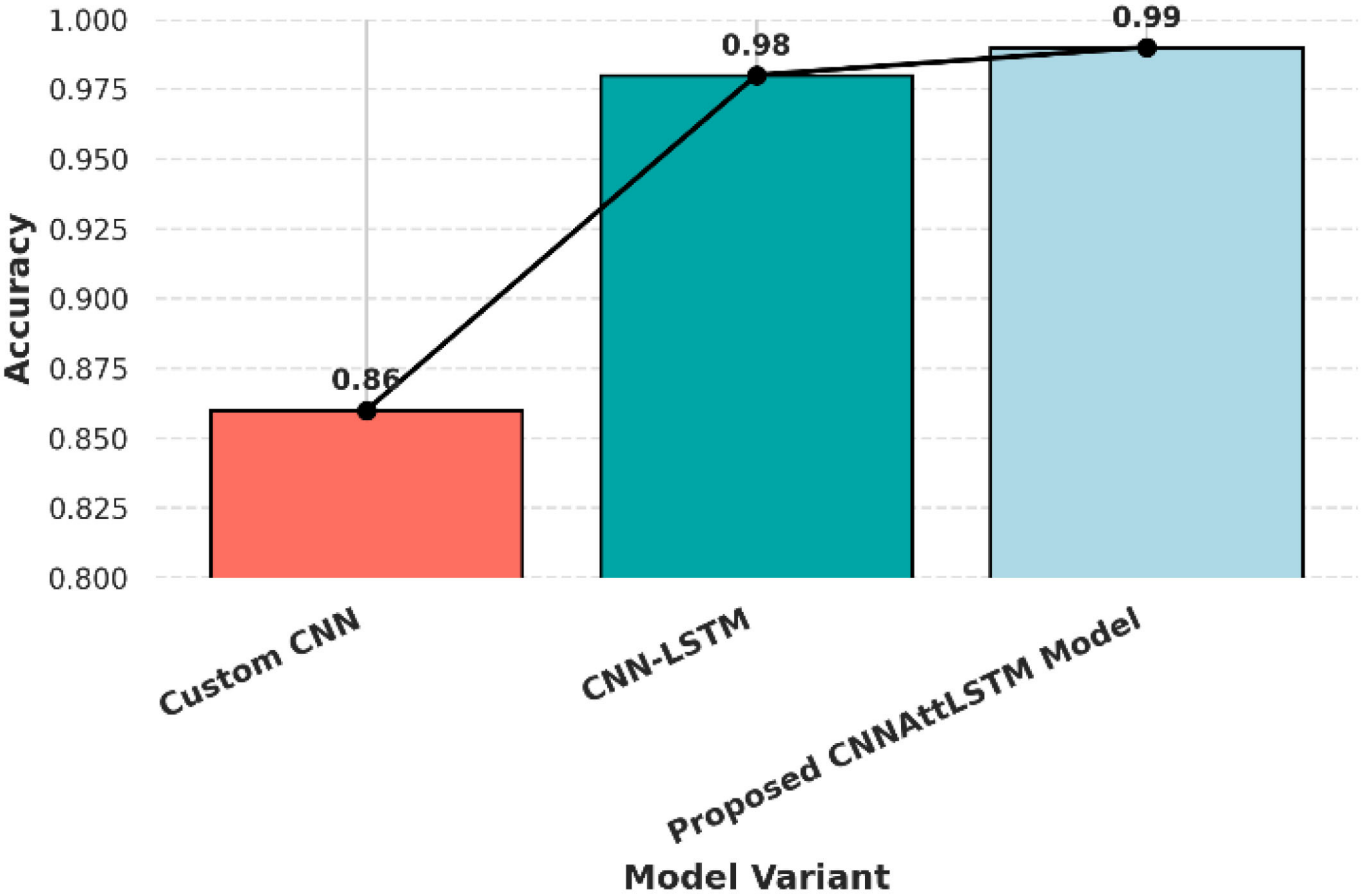
Comparative analysis of model performance accuracy.

To validate the contributions of key components in our CNNAttLSTM Model, systematically ablate LSTM and attention mechanisms, comparing performance against baselines in **Table 10**. The Custom CNN (86% accuracy) struggles with healthy leaf recall (0.32), while adding LSTM boosts F1-scores (0.96) but retains minor misclassifications. Integrating attention further refines results (99% accuracy, 1.00 recall for healthy leaves), confirming its role in feature refinement. **Figure 11** demonstrates the relative performance across different models.

**Table 10:** Ablation results.

Model variant	Accuracy	F1-score (Healthy)	Recall (Healthy)
Custom CNN	86%	0.45	0.32
CNN-LSTM (No Attention)	98%	0.96	0.99
Proposed CNNAttLSTM Model	99%	0.98	1.00

## 6 State-of-the-art comparison

A detailed comparison of the proposed CNNAttLSTM model with existing state-of-the-art methods is presented in **Table 11**. To comprehensively evaluate the robustness of various deep learning architectures for disease classification in jackfruit leaves, several state-of-the-art transformer-based deep models were applied to 38,019 images of the Jackfruit Leaf Disease Dataset. Results showed that early hybrid models like Hybrid CNN–Vision Transformer, which combined convolutional feature extraction with transformer-based contextual understanding, reported an accuracy of 81.30%. Then, later architectures such as the PMVT and SPT–LSA ViT further improved the performance to 87.60% and 88.57%, respectively, through the introduction of attention mechanisms together with localized feature representations. In addition, based on this self-attention technique, the Enhanced ViT reported an accuracy of 89.50%, while the Efficient Swin Transformer obtained an accuracy of 80% by employing hierarchical feature fusion. PLA-ViT marked a significant milestone in feature analysis with high precision for leaf features, obtaining an accuracy of 93%. Finally, the CNNAttLSTM integrates CNNs for spatial feature extraction, LSTM networks for learning sequential patterns, and attention mechanisms to focus on disease-relevant image sections and achieves the best performance of 99% accuracy. This really shows the exceptional capability of this model in capturing complex spatiotemporal relationships and fine-grained texture variations present within the jackfruit leaf images, outperforming state-of-the-art transformer-based models.

**Table 11:** Comparative analysis of state-of-the-art leaf disease classification models.

Ref.	Model	Technique	Performance metrics (%)
(De Silva and Brown, 2023)	Hybrid CNN–Vision Transformer	Convolutional Neural Networks (CNNs) + Vision Transformers (ViTs)	81.30%
(Li et al., 2023)	Plant-based Mobile Vision Transformer (PMVT)	Modified MobileViT Backbone + Convolutional Block Attention Module + Vision Transformer Encoder + Residual Fusion	87.60%
(Lye and Ng, 2023)	SPT–LSA ViT (Vision Transformer)	Vision Transformer + Shifted Patch Tokenization (SPT) + Locality Self-Attention (LSA)	88.57%
(Ali et al., 2025)	Enhanced Vision Transformer (ViT)	Vision Transformer (ViT) + Self-attention mechanisms	89.50%
(Zhang and Liu, 2025)	Efficient Swin Transformer	Swin Transformer + Selective Token Generator + Feature Fusion Aggregator	80%
(Murugavalli and Gopi, 2025)	PLA-ViT (Precision Leaf Analysis with Vision Transformers)	Vision Transformer (ViT) + multi-head self-attention	93%
Proposed CNNAttLSTM Model		CNN + LSTM + Attention Mechanism	99%

This work is unique in three important ways compared to existing studies. First a pseudo-temporal patch-based modelling strategy is introduced in which each image is decomposed into ordered 56×56 patches, allowing the LSTM to learn spatial–contextual relationships across leaf regions—an approach not used in previous jackfruit or plant disease classification works. Second, unlike prior models that rely solely on CNNs, transfer learning, or Transformer-based architectures, the proposed model uniquely integrates a lightweight CNN backbone, sequential modelling through LSTM, and a temporal attention mechanism within one framework, enabling selective emphasis on disease-critical patches. Third, while many existing SOTA models are computationally heavy, CNNAttLSTM achieves higher accuracy (99%) with only 3.7M parameters and 22 ms inference time, making it significantly more efficient and suitable for real-time and edge-device agricultural deployment. These aspects collectively distinguish this work from prior research.

## 7 Conclusion and future work

This study has thoroughly investigated three deep-learning models for classifying jackfruit leaf disease, demonstrating continuous performance improvement through sequential enhancements to the architecture. The first baseline model was a Custom CNN that achieved an accuracy of 86 per cent but was unable to classify healthy leaves (recall = 0.32) correctly. The addition of LSTM layers (CNN-LSTM) resulted in a significant improvement in accuracy to 98%, which was able to overcome the detection problem of healthy leaves (recall = 0.99). The CNNAttLSTM architecture has shown the best results with 99% accuracy and almost perfect classification in all categories and high precision (0.97) and F1-scores (0.98). The ablation analysis revealed that both the LSTM and attention components were relevant to these gains, with attention making a particularly significant contribution to the improvement in feature refinement and reduction of misclassification rates. Empirical evidence supports the concept that the joint use of CNN-based feature extraction and sequential modelling, along with attention mechanisms, can significantly increase the accuracy of plant disease detection. Future research suggestions consist of increasing the sample size and including more types of diseases and diverse environmental conditions to enhance generalisation; developing light-weight implementations of the model to deploy it in fields in agriculture; applying explainable AI model to provide interpretable results to the end-user; developing real-time monitoring platforms by combining the model with IoT sensors in the field; and modifying the architecture to other crops and to more critical plant health measurements. Additional edge computing and on-site testing optimisation in real-life agricultural settings would aid in proving the robustness and reducing the discrepancy between controlled-environment performance and applicability in the field, particularly in terms of precision agriculture. A notable limitation of this study is that the dataset used was entirely collected from jackfruit-growing regions within Bangladesh. Consequently, the model’s performance may vary under different environmental conditions, lighting setups, and disease manifestation patterns that occur in other geographical locations. Future work will focus on improving the model’s generalizability by retraining or fine-tuning the CNNAttLSTM architecture on region-specific datasets and by validating it against independent data collected from other jackfruit-producing countries such as India, Thailand, and Malaysia. Incorporating diverse climatic and ecological conditions will enable the model to learn broader disease features, thereby enhancing its adaptability for global agricultural use.

While the proposed CNNAttLSTM model is designed to be lightweight and suitable for real-time deployment on edge devices, several challenges must still be addressed, including limited on-board memory, lower computational throughput, restricted power budgets, and potential latency variations under field conditions. To mitigate these issues, the model can be further optimized using techniques such as quantization (8-bit or mixed precision), weight pruning, and model distillation to reduce parameter size and memory footprint without degrading accuracy. Additionally, deploying the model on hardware-efficient accelerators (e.g., NVIDIA Jetson Nano, Google Coral Edge TPU) and using optimized inference engines such as TensorRT or TFLite can significantly improve speed and energy efficiency. Offline caching of feature maps, batching strategies, and adaptive input resizing can also help overcome bandwidth and resource limitations. Therefore, although edge deployment presents inherent challenges, these can be effectively eradicated through targeted optimization strategies, ensuring the model’s practical usability in real agricultural settings.

## Data Availability

Publicly available datasets were analyzed in this study. This data can be found here: https://www.kaggle.com/datasets/shuvokumarbasak4004/jackfruit-leaf-diseases.
